# Brain Sex: Differences That Do Not Differentiate

**DOI:** 10.1007/s10508-025-03306-z

**Published:** 2025-10-10

**Authors:** Sallie Baxendale

**Affiliations:** Department of Clinical and Experimental Epilepsy, UCL Queen Square Institute of Neurology, London, WC1N 3BG UK

## Introduction

The use of neuroimaging to study transgender individuals has generated both clinical interest and societal attention. Identifying a “transgender brain” is seen by some as evidence of an enduring, innate gender identity. This appeals both to those who subscribe to the notion of a biologically determined gender identity and to clinicians charged with identifying the best treatment pathways for young people with gender-related distress. Others emphasize the influence of environmental and psychosocial factors on brain structure and function, suggesting that neuroimaging could help elucidate the factors that contribute to a trans identity.

Neuroimaging may present as an objective lens, but the interpretation of imaging data is shaped by a series of complex human decisions, making it highly susceptible to bias. Brain imaging studies are particularly vulnerable to misinterpretation in the highly polarized field of gender medicine, where those on all sides of the debate exhort others to follow the science. This commentary explores what the current neuroimaging literature can and cannot tell us about gender identity and proposes a framework for integrating neuroscience into gender research to ensure scientific rigor and ideological neutrality in this challenging research field.

## Neuroimaging Methods: Tools and Limitations

Neuroimaging methods can be broadly categorized into structural, functional, and molecular techniques, with additional distinctions based on the neural signal measured and temporal/spatial resolution of the data gathered. Each neuroimaging technique has its strengths and limitations as a tool to help us understand brain–behavior relationships. A summary of neuroimaging techniques is provided in Table 1.

**Table 1:** Summary of neuroimaging techniques

Neuroimaging type	Techniques	Measures	Strengths	Limitations
Structural imaging	MRI, CT	Brain anatomy (volume, thickness, integrity)	High spatial resolution; widely available	In the non-pathological brain, size and shape is poorly correlated with function or behavior
Functional imaging	fMRI (task-based, resting-state)	Brain activity via blood oxygen level dependent (BOLD) signal	Maps function; non-invasive	Low temporal resolution; indirect measure of function
Electrophysiological	EEG, MEG	Electrical/magnetic activity of neurons	High temporal resolution; real-time data	Poor spatial resolution; localization issues
Molecular imaging	PET, SPECT	Neurotransmitters, receptors, metabolism	Biochemical specificity; unique insights	Invasive, Radiation exposure; expensive; low resolution
Connectivity imaging	DTI, functional/ effective connectivity	White matter tracts, network correlations	Reveals brain networks and pathways	Correlational data; complex modeling
Multimodal imaging	PET-MRI, EEG-fMRI	Multiple signal types (e.g., structure + function)	Integrates strengths of individual methods	Technically complex; potential artifacts

## Neuroimaging in Transgender Research

Neuroimaging studies of transgender individuals have primarily aimed to identify structural or functional correlates of a trans identity, gender dysphoria, or the effects of hormonal therapy.

### Structural Imaging

Using structural neuroimaging to determine whether transgender individuals are “born in the wrong body” is fundamentally flawed, since the brain is not a sexually dimorphic organ. Unlike the binary nature of reproductive anatomy, no structural feature of the brain definitively identifies it as male or female. Although group differences in brain volumes between males and females have been reliably reported in large-scale studies (Flint et al., 2020; Guillamon et al., 2016; Luders et al., 2009; Ritchie et al., 2018; Ruigrok et al., 2014), there is considerable overlap between the sexes. The differences reported are differences that do not differentiate.

However, the whole can be more than the sum of its parts. Machine learning models can detect subtle patterns across thousands of features and can differentiate between male and female brains with a level of accuracy well above chance (in the range of 85–93%) (Anderson et al., 2019; Dibaji et al., 2024; Ebel et al., 2023). While these rates may initially seem impressive, high levels of accuracy can also be obtained on the basis of height alone. For example, using U.S. adult height data, if everyone taller than 168.5 cm was classified as male and everyone shorter as female, approximately 83% of the US population would be correctly classified.^1^

The male/female overlap in brain structure is substantial and, just as in height, the differences identified in these studies may be statistically significant at a group level, but cannot be used as a categorical marker at an individual level (Joel & Tarrasch, 2014). Just as a female is not more male if she is 169 cm tall, a brain is not less male if it has some structural characteristics that are more common in females. The significant overlaps in the data tell us something about our measure, i.e., that it is not sex specific, rather than the maleness/femaleness of the brain. While group differences may be statistically significant, if substantial numbers of males and females share the same characteristic, that characteristic is not a sex specific marker.

### Functional Imaging

Functional neuroimaging offers more promise in exploring the mechanisms underlying transgender identity. These methods can reveal patterns of brain activity at rest or during tasks, highlighting regions that are atypically connected or activated.

Studies have reported differences in activation in transgender individuals across several brain regions, including the visual cortex, the medial superior frontal gyrus, the supplementary motor area, the lingual gyrus, the putamen, the precuneus, the anterior and posterior cingulate cortex, and the ventral striatum (Frigerio et al., 2021; Kim et al., 2016; Majid et al., 2020) (Table 2).

**Table 2:** Brain regions implicated in imaging studies of transgender people

Brain region	Associated functions
Medial superior frontal gyrus	Self-referential processing, decision making, and aspects of social cognition
Supplementary motor area	Planning and coordination of movement, motor imagery, and response inhibition
Lingual gyrus	Visual processing, particularly related to letters and complex images; involved in encoding visual memories
Putamen	Motor control, learning, reward processing, and habit formation
Precuneus	Self-consciousness, visuospatial imagery, episodic memory retrieval, and aspects of consciousness
Posterior cingulate cortex	Autobiographical memory, self-reflection, and integration of emotional and cognitive information
Anterior cingulate cortex	Emotion regulation, conflict monitoring, decision making, and error detection
Ventral striatum	Reward processing, motivation, reinforcement learning, and hedonic experience

While these studies may point to potential neural correlates of gender dysphoria or a trans identity, they do not establish causality. Neither are these patterns unique to transgender individuals. These regions are also implicated in a wide array of psychiatric and neurodevelopmental conditions. For example, the anterior cingulate cortex has been implicated in studies of depression, anxiety disorders, schizophrenia, obsessive–compulsive disorder, ADHD, chronic pain, and substance use disorders. Brain regions associated with self-perception have also been implicated in studies of patients with other forms of body dysmorphia, such as those with anorexia.

## P-Hacking in the Brain: A Playground for False Positives

Neuroimaging analyses involve multiple preprocessing decisions in the treatment of tens of thousands of voxels, creating many “researcher degrees of freedom” (Simmons et al., 2011). Different preprocessing pipelines or statistical thresholds can yield conflicting results from the same dataset. This flexibility makes imaging studies particularly vulnerable to p-hacking—adjusting analyses until results become statistically significant, even if the patterns are spurious. As a result, some reported “activations” can mean little more than what the researchers chose to highlight. This was illustrated in the infamous dead salmon experiment (Bennett et al., 2009). In the first and only study reported in the *Journal of Serendipitous and Unexpected Results*, researchers placed a dead fish in an fMRI scanner and showed the fish images of people in social situations, instructing it to identify their emotions. Significant brain activity was detected. The paper has become a totemic case study demonstrating how poor statistical controls can lead to false positives in neuroimaging studies. There are growing concerns about the failure to replicate a very high proportion of functional imaging studies, not just in the field of gender medicine (Lyon, 2017). Without preregistration or transparent methodology, neuroimaging findings can be tailored—consciously or not—to support a preferred narrative. This issue is compounded in gender research, where social and political pressures, and predetermined convictions may strongly influence interpretation.

## Methodological Best Practices: Toward Scientific Rigor

In studies exploring the “transgender brain,” additional complexities arise. Study designs need to control for a multitude of confounding factors, including neurodevelopmental stage, psychiatric comorbidities, sexual orientation, and neurodiversity. Without such controls, it becomes very challenging to isolate brain differences that specifically relate to gender identity.

Controlling for hormonal exposure is particularly important. The brain contains numerous sex hormone receptors—primarily estrogen receptors (ERα and ERβ) and androgen receptors—which are heavily concentrated in areas such as the hypothalamus, amygdala, and hippocampus. These regions are key for regulating sexual behavior, emotional responses, aggression, libido, and mood.

Notwithstanding overlaps, the distribution and density of these receptors differ between the sexes. When the brain’s hormonal "fuel" mix changes—such as during puberty or with the introduction of cross-sex hormones—behavior changes too. Focusing only on the brain’s structure, while ignoring the hormonal fuel it runs on, can only provide a partial understanding of the neurological factors that shape gender dysphoria or a trans identity.

To maximize their value, future neuroimaging studies in the field of gender identity should adhere to rigorous design principles. These include: (1) the use of pre-registered, hypothesis-driven protocols; (2) sufficiently large and statistically powered sample sizes. Inadequately powered studies increase the risk of false positives and produce inflated effect sizes. A priori power analyses should be reported as part of study design, using effect sizes drawn from prior studies or meta-analyses to ensure appropriate sample estimates; (3) masked (blinded) analyses with appropriate corrections for multiple comparisons; (4) careful control for confounding variables such as hormonal treatment/exposure, psychiatric comorbidities, and developmental stage; (5) inclusion of drug-naïve participants whenever feasible; and (6) stratified analyses based on biological sex, sexual orientation, and neurodiversity. Adhering to these standards will enhance reproducibility, reduce bias, and increase the clinical and scientific value of research in this complex and evolving area. Even in the context of robust statistical findings, researchers should avoid conflating statistical significance with clinical relevance.

## Interpreting Brain Differences: Beyond Reductionism

Neuroimaging provides insights into the correlates—not causes—of gender dysphoria. The physical brain is a result of a dynamic interplay of biological, psychological, and socio-environmental factors. Overemphasizing structural or functional brain differences risks a form of “neuroessentialism,” attempting to locate identity in immutable neural networks and pathways. Rather than seeking a signature “transgender brain,” neuroscience should contribute to a broader understanding of gender diversity. Used carefully, neuroimaging can be an invaluable tool to enrich our understanding—but it must inform, not define, the complex realities of a trans identity.

## Footnotes

1 Based on USA data, male and female height distributions differ with a mean average of 175.3 cm (± 7.4 cm) in males and 161.6 cm (± 7.1 cm) in females. The two distributions intersect at 168.5 cm. Based on these distributions, 82.2% of males fall above this threshold, while 83.3% of females fall below it. Assuming an equal number of males and females in the population, the overall classification accuracy of males and females is around 82.75% using 168.5 cm as a cutoff.
